# Knockdown of IFI27 inhibits cell proliferation and invasion in oral squamous cell carcinoma

**DOI:** 10.1186/s12957-018-1371-0

**Published:** 2018-03-27

**Authors:** Hong Wang, Xunding Qiu, Shigeng Lin, Xubin Chen, Tao Wang, Tianan Liao

**Affiliations:** Oral and Maxillofacial Surgery, Hainan General Hospital, Xiuhua Road NO.19, Xiuying District, Haikou, 570311 Hainan China

**Keywords:** Oral squamous cell carcinoma, IFI27, Apoptosis, Tumor invasion

## Abstract

**Background:**

The development of oral squamous cell carcinoma (OSCC) involves genetic mutations, epigenetic gene expression modification, and other processes. It has been reported that IFI27 is upregulated in OSCC, but its function is unknown. The aim of this study was to investigate the role of IFI27 on OSCC cell proliferation and invasion.

**Methods:**

The protein level of IFI27 in OSCC tissues and adjacent tissues was detected by immunohistochemistry. In the OSCC cell model, we designed the IFI27 siRNA to downregulate the expression of IFI27; gene and protein of IFI27 in those models were then detected by Q-PCR and Western blot. MTT assay was used to detect the effect of -IFI27 knockdown on cell proliferation; Annexin V-PI staining flow cytometry was used to detect the effect of IFI27 downregulation on apoptosis of cancer cells. The effect of IFI27 downregulation on oral cancer cell invasion was detected using Transwell assay.

**Results:**

IFI27 was highly expressed in OSCC tissues by immunohistochemical assay. In the OSCC cell model, IFI27 siRNA could downregulate the mRNA and protein expression level of IFI27. As showed in MTT assay, Annexin V-PI assay, and Transwell assay, through the downregulation of IFI27, TSCCA and TCA8113 cell proliferation were inhibited, OSCC cell apoptosis was promoted, and its migration and invasion were inhibited.

**Conclusion:**

IFI27 is involved in the development and progression of OSCC. Its high expression promotes cell proliferation and invasion and reduces apoptosis. These findings may provide new biomarkers and therapeutic targets for OSCC diagnosis and clinical treatment.

## Background

Head and neck squamous cell carcinoma (HNSCC) is one of the top ten most fatal cancers in the world. Statistics has shown that in the USA alone, the annual number of new HNSCC patients can reach as many as 49,670 cases, of which oral squamous cell carcinoma (OSCC) patients accounted for about 65% [1]. The occurrence and development of OSCC is a multifactorial, long-term process. Individual genetic variation, environmental factor stimulation (such as alcohol and tobacco), and viral infection (human papillomavirus) are considered to be associated with the onset of OSCC [2, 3]. The current clinical treatments of OSCC mainly include surgery, radiotherapy, and chemotherapy, but for advanced patients, because of its rapid deterioration of the disease, these traditional treatments have little effect [4]. Therefore, further exploring the basis of OSCC molecular mechanisms to find new markers for the early diagnosis of OSCC has become particularly important.

Interferon alpha-inducible protein 27 (IFI27) is a hydrophobic mitochondrial protein composed of 122 amino acid [5]. Rosebeck and Leaman et al. reported that IFI27 maintains a low background expression in a variety of mammalian cells and participates in multiple biological processes, including apoptosis and congenital immunity [6, 7]. However, IFI27 expression was confirmed to be elevated in the psoriatic lesions and uterine fibroids, ovarian cancer, and other diseases [7–10]. Skov et al. have shown that IFI27 was highly expressed in sera of patients with primary myelofibrosis compared with normal subjects and that their expression levels were consistent with the severity of the disease [11]. It was reported that in a variety of cancers, the downregulation of IFI27 affects tumor development. For example, downregulation of IFI27 can promote TRAIL-induced apoptosis in animal and cellular models of hepatocellular carcinoma and gastric cancer, suggesting that IFI27 may be a key protein for tumor development [12]. These studies suggest that IFI27 can be used as a diagnostic indicator of cancer and a potential therapeutic target.

In this study, we aim to determine the expression level of IFI27 in OSCC and to investigate its effect on OSCC cell proliferation and invasion, providing new markers and therapeutic targets for OSCC diagnosis and clinical treatment.

## Methods

### Sample preparation and cell culture

Tongue pathological tissue and adjacent tissue samples were collected from 25 OSCC patients at the Department of Stomatology in the Hainan Provincial People’s Hospital between January 2013 and January 2017. All biopsies were diagnosed with either well-differentiated or moderately differentiated OSCC. Written informed consent was obtained for each participant, and the study was approved by the Hospital Ethics Committee.

OSCC cell lines TSCCA and TCA8113 were purchased from the cell bank of the Chinese Academy of Sciences; cells were cultured with RPMI-1640 (Gibco) medium containing 10% fetal bovine serum (FBS) and 1% streptomycin (penicillin-streptomycin solution, PS).

### Immunohistochemistry

OSCC and adjacent tissue samples were paraffin embedded and sliced into 5-μm sections, dewaxed with xylene, and rehydrated in concentration gradient ethanol solutions. For antigen retrieval, samples were placed in citrate buffer (10 mM, pH 7.0) in a warm water bath. The sample sections were then blocked with 10% sheep serum for 30 min at room temperature. The IFI27 primary antibody were added to the sections and incubated overnight at 4 °C. Detection was performed using a secondary antibody incubation at RT for 1 h, followed by colorimetric detection using DAB. Tissues were counterstained with hematoxylin and mounted with neutral resin sheet. All samples were carefully observed under a laser confocal microscopy at × 400 magnification (XSP-C204, COIC, Chongqing, China).

### IFI27 siRNA transfection

OSCC cells were cultured in six-well plates for 12–24 h until the cell density reached 60–70%. After rinsing twice with serum-free RPMI-1640 solution, IFI27 siRNA was transfected using the Lipofectamine™ RNAiMAX (invitrogen) kit, following the manufacturer’s instructions. The siRNA sequences are described below. After transfection, cells were cultured in RPMI-1640 medium containing 10% FS and subjected to subsequent experiments.

**Table:** 

siRNA1	5′-GCUGUCAUUGCGAGGUUCU-3′ (sense),
	5′-AGAACCUCGCAAUGACAGC-3′ (antisense),
siRNA2	5′-GCCCUGCAGAGAAGAGAAC-3′ (sense),
	5′-GUUCUCUUCUCUGCAGGGC-′ (antisense).

### RNA extraction and real-time quantitative PCR

Total RNA was extracted from TSCCA and TCA8113 cells using TRIzol (Invtrogen). Two micrograms of RNA was reverse transcribed using the First Strand cDNA Synthesis Kit (TOYOBO). Real-time quantitative PCR was performed using the 7900 HT Fast, the Ct values were obtained, and 2^-ΔΔ Ct^ were calculated. The primers used in this study were:

**Table:** 

GAPDH-F	5′-CAAGAAGGTGGTGAAGCAGGC-3′
GAPDH-R	5′-CATACCAGGAAATGAGCTTGAC-3′
IFI27-F	5′-CTTCACTGCGGCGGGAATC-3′
IFI27-R	5′-CCAGGATgAACTTGGTCAATCC-3′

### Western blot analysis

The sample cells were cleaved using RIPA (50 mM Tris, 150 mM NaCl, 1% NPNP-40, 1% sodium deoxycholate, and 0.05% SDS, pH 7.4). Proteins were quantified using the BCA kit (Bio-Rad Laboratory, Hercules, CA, USA). The protein loading was 40 μg. After electrophoresis and membrane transferring, the membrane was incubated at 4 °C overnight with first antibody (anti-IFI27, 1: 3000); the secondary antibody was then incubated at room temperature for 1 h, followed by coloration and protein analysis.

### Cell proliferation assay (MTT)

Twenty-four hours after the transfection, the cells were digested and washed, the cell concentration was then adjusted to 1 × 10^5^ cells/mL, and 100 μL was added into each well of a 96-well plate, i.e., 1 × 10^4^ cells/well. After incubation for 24, 48, and 72 h, 1/10 volume of MTT (50 nM) solution was added and then incubated for another 4 h. Add 100 μL DMSO to each well and shake it slowly and gently for 10 min at 37 °C in the dark. The absorbance was then measured at 570 nm. Apoptotic TSCCA and TCA8113 cells were analyzed in triplicates and repeated three times independently.

### Flow cytometric analysis of apoptosis

Cells were harvested 48 h after transfection, and 5 × 10^5^ cells were resuspended in 200 μL of RPMI-1640 serum-free medium. 1.25 μL of Annexin V-FITC was added and incubated at room temperature (18–24 °C) in darkness. After 15 min, it was centrifuged and the supernatant were removed. The cells were gently resuspended with 0.5 mL of pre-cooled 1× binding buffer, and 10 μL of propidium iodide was added. The sample was then placed on ice and in darkness, and flow cytometry assay (Beckman Coulter) was performed.

### Cell migration assay

1 × 10^5^ cells were resuspended in 100 μL of serum-free medium and added to the upper chamber of Transwell (8-μm pore size; BD Biosciences, San Jose, CA, USA), and 600 μL of complete medium was added to the lower chamber. After incubated for 24 h at 37 °C and 5% CO_2_, remove the upper chamber and wipe the cells from it with a cotton swab. Four percent paraformaldehyde was added, and samples were fixed for 15 min. Washed with PBS both before and after crystal violet staining, and then, pictures were taken and analyzed.

### Cell invasion assay

Matrigel was dissolved overnight at 4 °C. The Matrigel solution was then diluted in 1:3 volume ratio with pre-cooled serum-free medium; 40 μL was added to the pre-cooled Transwell chamber and incubated at 37 °C for 2 h for coagulation. Remove the excess liquid in the chamber, add 100-μL serum-free medium into the upper chamber, and add 600-μL medium into lower chamber, respectively, balanced overnight at 37 °C. After transfected with siRNA for 48 h, 1 × 10^5^ cells were resuspended in 100-μL serum-free medium. The suspension were added to the upper chamber of Transwell, and 600 μL of complete medium was added to the lower chamber. After incubated for 48 h at 37 °C and 5% CO_2_, remove the upper chamber and wipe the cells from it with a cotton swab. Samples were then fixed in 4% paraformaldehyde for 15 min, washed with PBS prior to and after crystal violet staining. The result was photographed and analyzed.

### Data analysis

All data were analyzed by SPSS13.0 statistical analysis software, enumeration data expressed in %, with *χ*^2^ test for comparison. Measurement data were expressed as $\overline{x}\pm s$, with paired *t* test for comparison between the groups. For non-normal distribution data and data with uneven variances, rank-sum test was used. The statistical significance was set at *P* < 0.05.

## Results

### IFI27 was highly expressed in OSCC

**Fig. 1:**
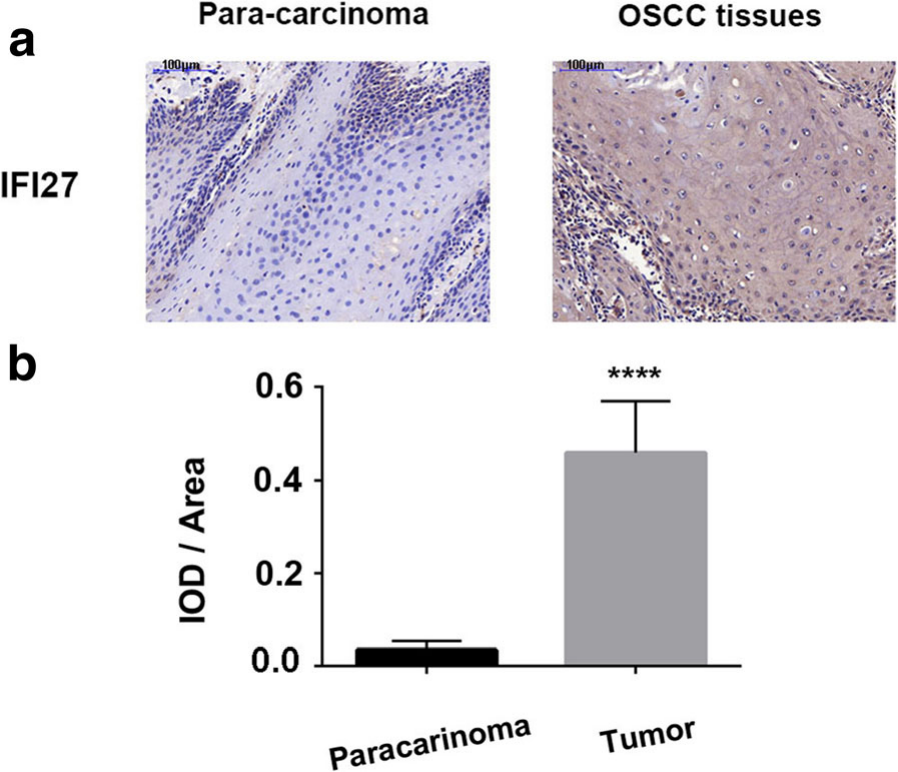
IFI27 was upregulated in OSCC tissues. Representative immunohistochemistry staining of IFI27 in 25 patients with OSCC and para-carcinoma tissues (**a**) and the statistic results of IOD/area (**b**). ****p < 0.0001 compared with negative control (NC) group

We recruited 25 patients with OSCC; tumor tissue and its adjacent tissues were collected from each one of them. Immunohistochemical analysis was performed to study the expression of IFI27 in OSCC. Results of the staining were shown in Fig. 1; the staining of IFI27 protein was lower in normal tissues; however, it was significantly increased in OSCC tissues, suggesting that IFI27 is highly expressed in OSCC (Fig. 1a, b).

### The downregulation of IFI27 by siRNA in OSCC cell lines

**Fig. 2:**
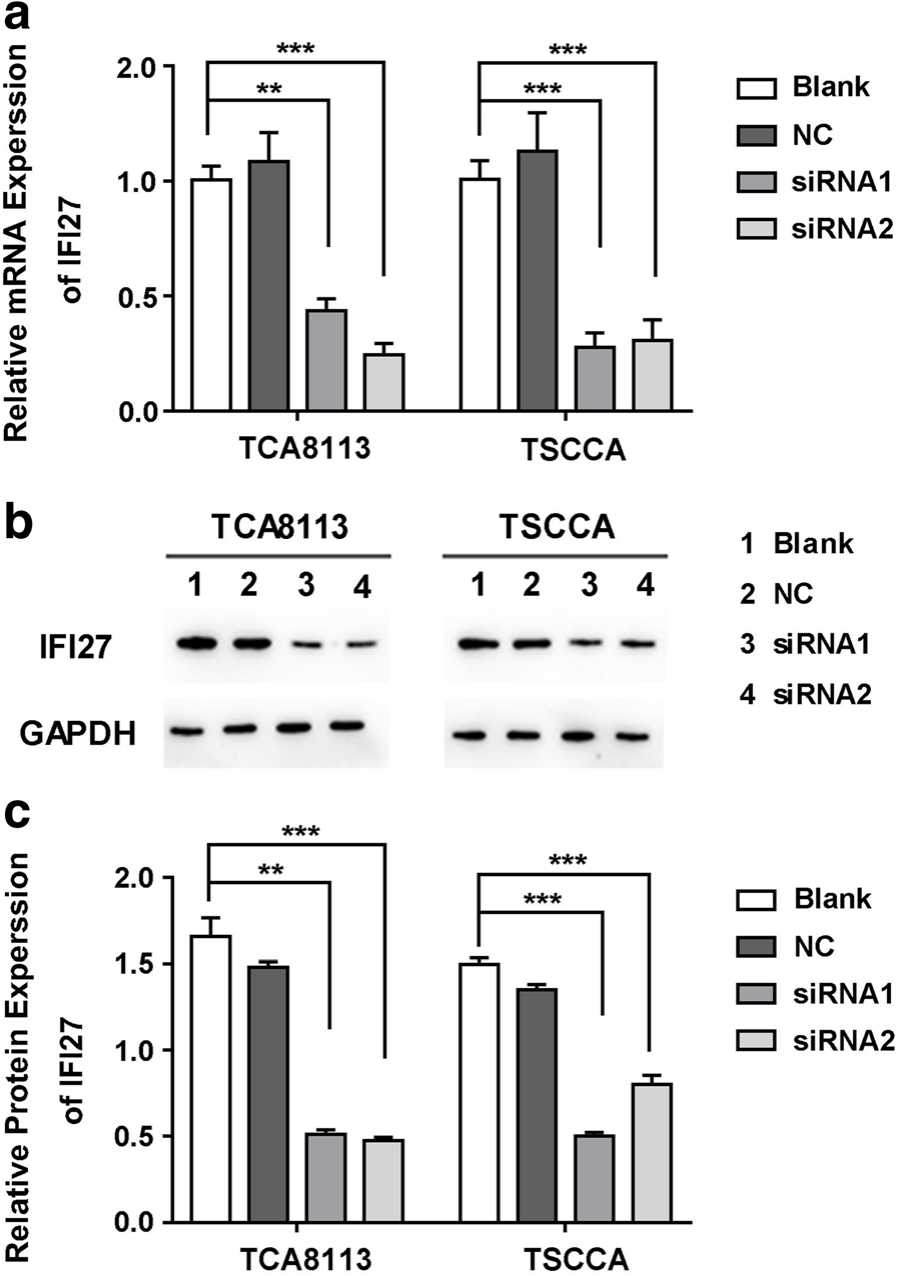
The IFI27 expression levels in the siRNA transfected OSCC cells. Relative mRNA levels (**a**, **c**) and protein levels (**b**) of IFI27 in TCA8113 and TSCCA cells transfected with negative control, siRNA1, or siRNA2. **p < 0.01, ***p < 0.001 compared with NC groups

To further study the role of IFI27 in oral squamous cell carcinoma, two siRNA sequences targeting IFI27 were designed and transfected into TSCCA and TCA8113 cells. The IFI27 mRNA and protein levels were detected by qRT-PCR and Western blot respectively. As shown in Fig. 2, qRT-PCR results showed that IFI27 mRNA expression levels in TSCCA and TCA8113 cells were significantly decreased after the transfection of siRNA1 or siRNA2 compared with blank control group (Fig. 2a); the level of IFI27 protein in those cells was also downregulated, according to Western blot results (Fig. 2b, c). These data suggested that the two siRNA sequences we designed can effectively knock down the expression of IFI27 in OSCC cells.

### Downregulation of IFI27 inhibits OSCC cell proliferation and promotes apoptosis

**Fig. 3:**
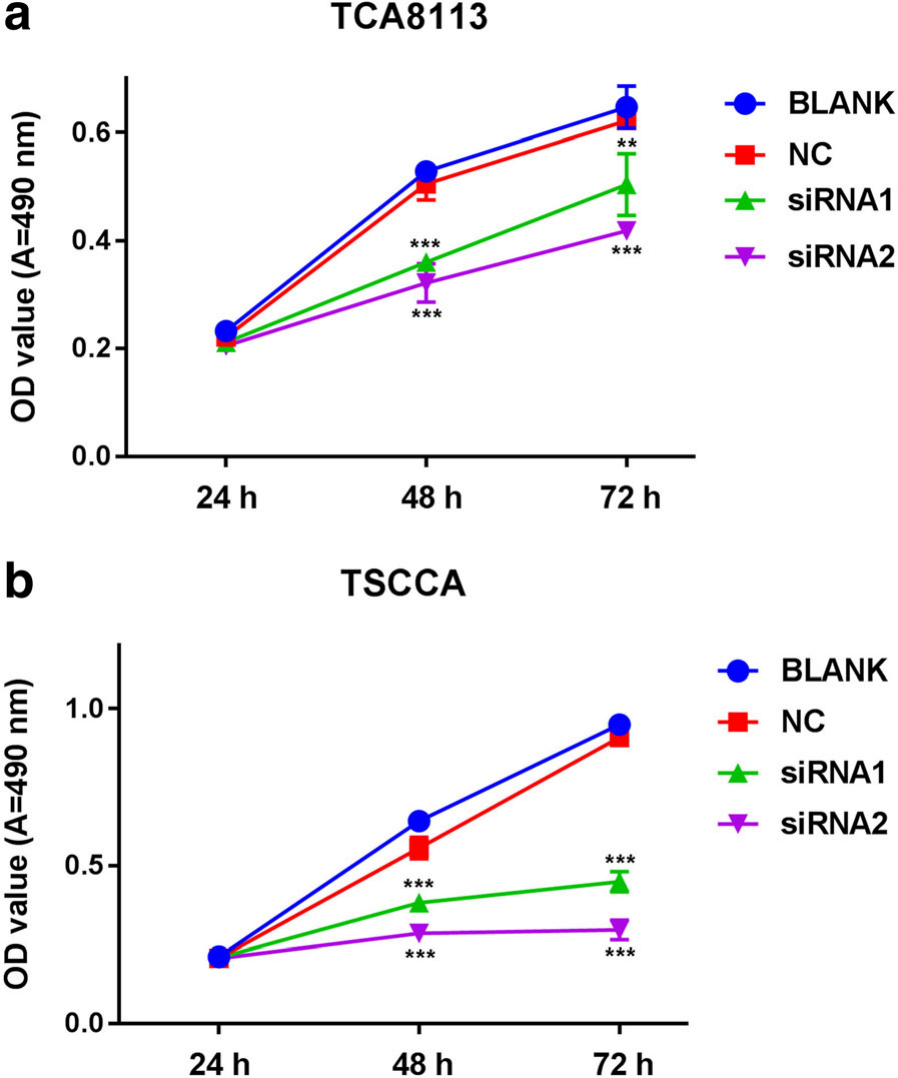
Suppression of cell proliferation after IFI27 knockdown. The transfected TCA8113 (**a**) and TSCCA cell (**b**) proliferation for each group (negative control, siRNA1 group, or siRNA2 group) measured by MTT after transfected with 24, 48, and 72 h. **p < 0.01, ***p < 0.001 compared with NC groups

**Fig. 4:**
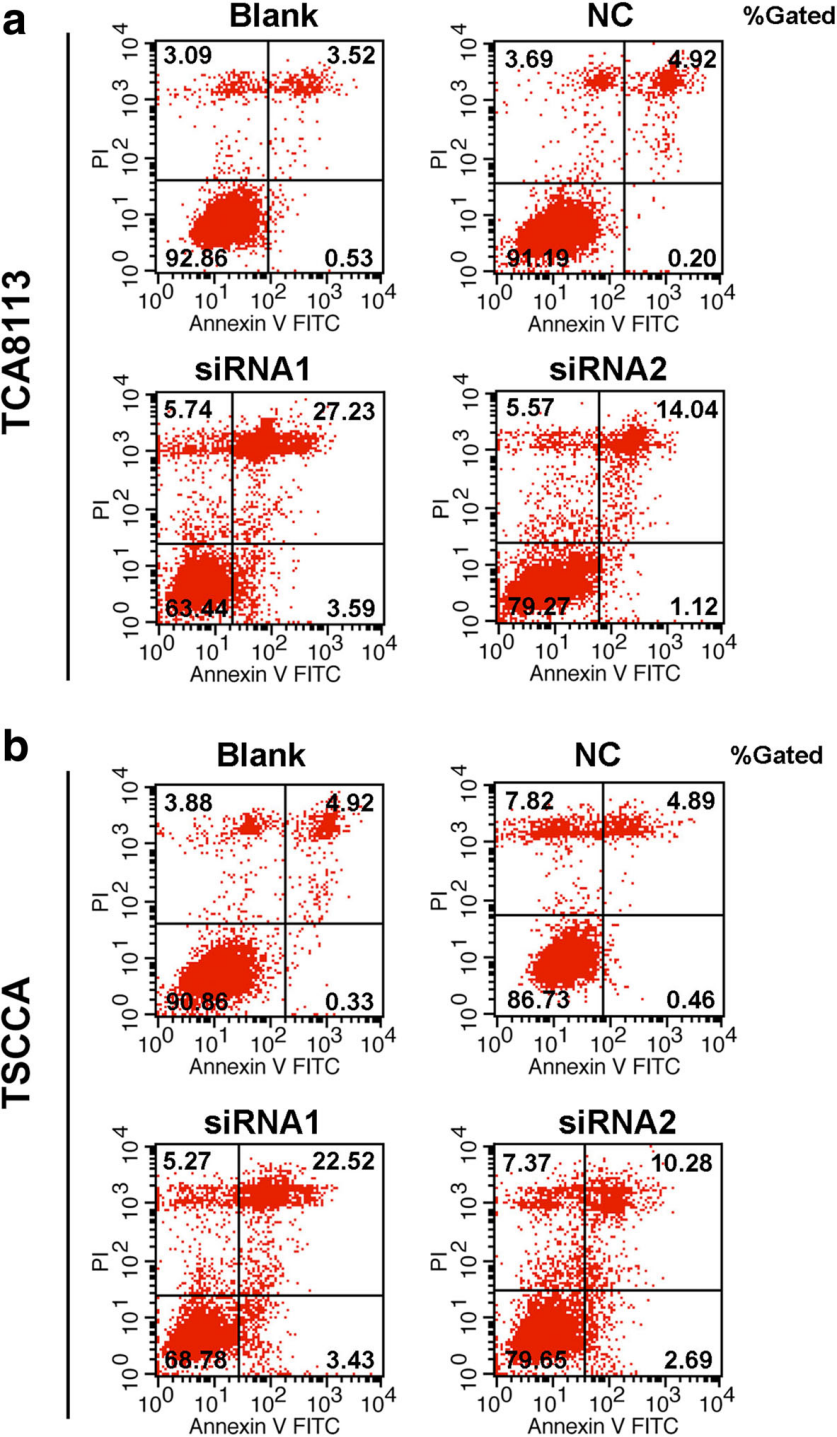
Effects of IFI7 knockdown on OSCC cells apoptosis. Flow cytometry was performed to measure the apoptosis ratio of TCA8113 cells (**a**) and TSCCA cells (**b**) transfected with negative control, siRNA1, or siRNA2

As described above, we downregulated the expression of IFI27 in two OSCC cell lines, TSCCA and TCA8113. MTT and flow cytometry were used to detect the effect of IFI27 downregulation on cell proliferation and apoptosis. When the expression of IFI27 in those two cancer cell lines was downregulated, the cell proliferation was apparently suppressed compared with the blank control group (Fig. 3a, b), according to the MTT results. Samples were Annexin V/PI stained and detected by flow cytometry, and it showed that compared with the blank control group, the percentage of apoptotic cells increased remarkably in the IFI27 downregulated group (Fig. 4a, b). These data suggest that downregulation of IFI27 expression in OSCC cancer cells can significantly inhibit OSCC cancer cell proliferation and promote its apoptosis.

### IFI27 downregulates OSCC cell migration and invasion

**Fig. 5:**
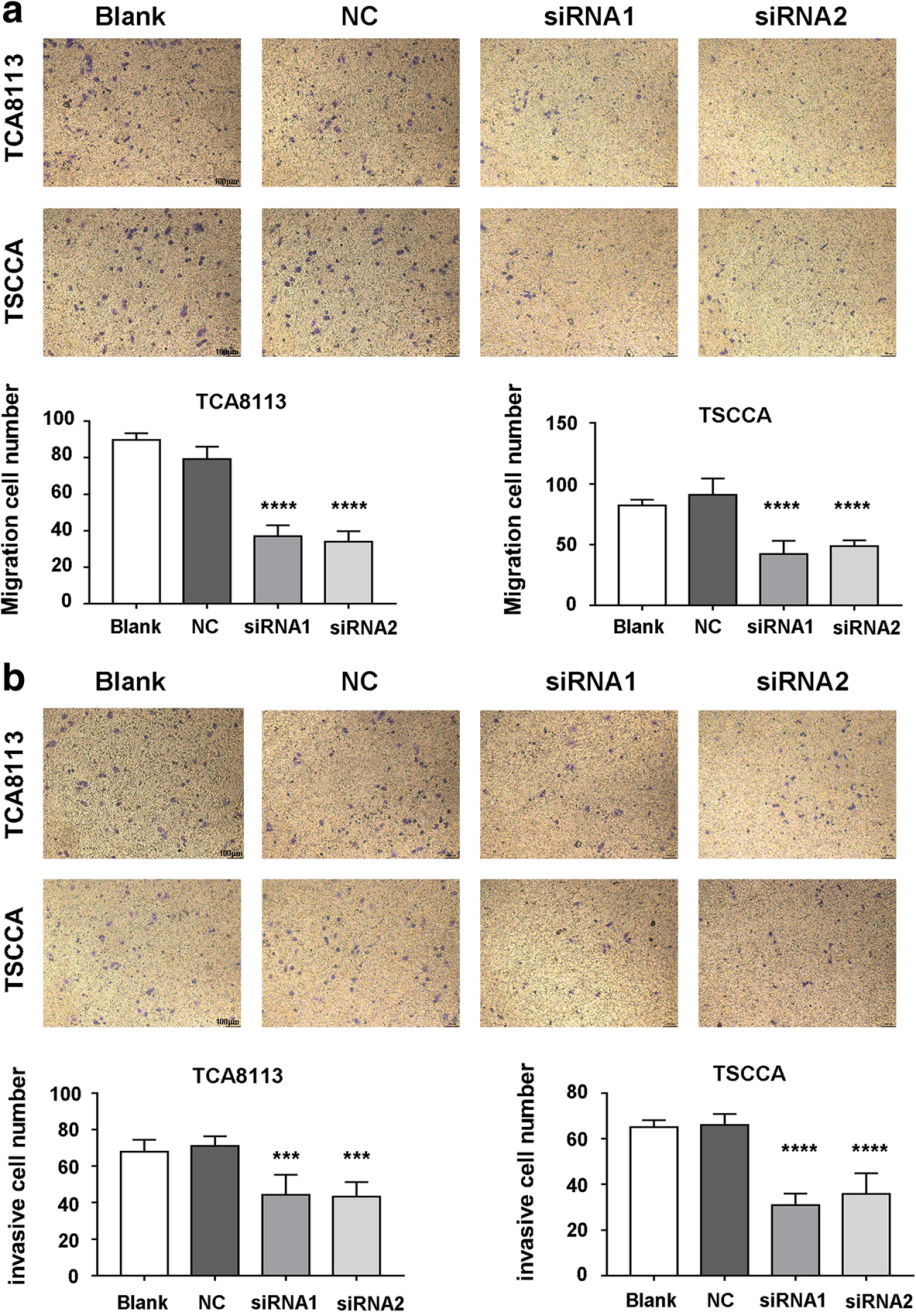
The migration and invasion abilities in the different cell groups. Transwell assays were performed to detect the effects of IFI27 knockdown on the migration (**a**) and invasion (**b**) ability of TCA8113 cells and TSCCA cells transfected with negative control, siRNA1, or siRNA2. ***p < 0.001 compared with NC group

The migration and invasion of cancer cells is particularly important for cancer progression. To clarify whether IFI27 was involved in the migration and invasion of OSCC cancer cells, we used Transwell assay. Crystalline violet staining results showed that the downregulation of IFI27 in TSCCA and TCA8113 cells resulted in a notable reduction in the number of cell migration and invasion compared to the control group (Fig. 5a, b). These results suggested that downregulation of IFI27 expression in OSCC cells can inhibit the migration and invasion of cancer cells.

## Discussion

Our study found that in patients with OSCC, the expression of IFI27 was apparently increased in cancer tissues. When siRNA was used to knock down the expression level of IFI27 in OSCC cell lines TSCCA and TCA8113, it was showed that the downregulation was able to inhibit cell proliferation, promote cell apoptosis, and inhibit cell migration and invasion, indicating that IFI27 was involved in OSCC occurrence and development.

The oral squamous cell carcinoma formation process, first of all, is the occurrence of precancerous lesions such as oral leukoplakia (OLK), followed by low degree of abnormal proliferation, and then the development of highly abnormal hyperplasia, and ultimately the formation of invasive malignant tumors [13]. Lymph node cytology, histopathological analysis, and saliva and serological analysis are commonly used methods for OSCC diagnosis [14, 15]. In recent years, several markers have been found for OSCC diagnosis. Xu et al. indicated that DJ-1 was upregulated in OSCC and may be involved in promoting OSCC proliferation and invasion [16]. Xue et al. revealed that HoxB9, by activating TGF-β/Slug signaling pathway, promotes OSCC cell migration and invasion [17]. However, there is still a lack of specific targets that can be used for clinical diagnosis, or for drug therapy.

IFI27 is one of the members of the interferon alpha-induced protein family, and it has been reported to be involved in IFN-induced apoptosis, cell proliferation, and immune responses. The upregulation of IFI27 was first reported in the psoriasis model, and in recent years, it has been found that IFI27, together with LCN2, can be used as two basal breast cancer subtype markers [18, 19]. In addition, Siegel et al. found that downregulation of IFI27 leads to a reduction in the formation of the cyclin A/CDK1 complex, resulting in epithelial cells stagnated in the S phase, thereby inhibiting cell proliferation [8]. Recently, Li et al. employed microarray analysis to screen the genes that were related to OSCC and revealed that five genes were highly expressed, including CXCL10 and IFI27 [20]. Boldrup et al. discovered that the expression of IFI27 in tongue squamous cell carcinoma and adjacent tissues were elevated, while it maintains a low background expression in normal tissues [21]. In this study, we observed that IFI27 was highly expressed in tissue samples of OSCC patients.

On the basis of these findings, we used the OSCC cell models to study the biological function of IFI27 for the first time. With RNA interference technology, our results showed that downregulation of IFI27 in TSCCA and TCA8113 cell lines can inhibit OSCC cell proliferation and migration and invasion and promote OSCC cell apoptosis. The exact molecular mechanism of IFI27 in OSCC and its role in vivo remains to be further explored.

## Conclusions

In conclusion, our data indicate that IFI27 is overexpressed in OSCC and is involved in OSCC cell proliferation, apoptosis, and invasion. These data provide possible diagnostic criteria and therapeutic targets for OSCC.
